# Introducing ‘Ngaruroro’, a New Model for Understanding Māori Wellbeing

**DOI:** 10.3390/ijerph21040445

**Published:** 2024-04-05

**Authors:** Finley Ngarangi Johnson, Priscilla Wehi, Tia Neha, Mike Ross, Veronica Thompson, Stephanie Tibble, Natasha Tassell-Matamua, Kevin Shedlock, Ririwai Fox, Zachary Penman, Tyler Ritchie, Taylor Winter, Hitaua Arahanga-Doyle, Paul E. Jose

**Affiliations:** 1 School of Psychology, Victoria University of Wellington, Wellington 6140, New Zealand; tia.neha@vuw.ac.nz (T.N.); tyler.ritchie@vuw.ac.nz (T.R.); paul.jose@vuw.ac.nz (P.E.J.); 2 Centre for Sustainability, University of Otago, Dunedin 9016, New Zealand; priscilla.wehi@otago.ac.nz; 3 Te Kawa a Māui—School of Māori Studies, Victoria University of Wellington, Wellington 6140, New Zealand; mike.ross@vuw.ac.nz; 4 Morehu Māori Basketball, Wellington 6022, New Zealand; 5 Tuahuru Marae, Te Māhia 4165, New Zealand; 6 School of Psychology, Massey University, Palmerston North 4442, New Zealand; n.a.tassell-matamua@massey.ac.nz; 7 School of Engineering and Computer Science, Victoria University of Wellington, Wellington 6140, New Zealand; kevin.shedlock@vuw.ac.nz; 8 School of Psychology, University of Waikato, Hamilton 3216, New Zealand; ririwai.fox@waikato.ac.nz; 9 Ministry of Social Development, Wellington 6140, New Zealand; zach.penman001@msd.govt.nz; 10 School of Mathematics and Statistics, University of Canterbury, Christchurch 8041, New Zealand; taylor.winter@canterbury.ac.nz; 11 Department of Psychology, University of Otago, Dunedin 9016, New Zealand; hitaua.arahanga-doyle@otago.ac.nz

**Keywords:** Māori, Kaupapa Māori, Māori wellbeing, Indigenous health, Indigenous wellbeing, qualitative research, thematic analysis

## Abstract

Indigenous peoples around the world are revitalising their ancestral beliefs, practices, and languages, including traditional understandings of health and wellbeing. In the Aotearoa (New Zealand) context, a number of ground-breaking Māori health- and wellbeing-related models have emerged, each with their own scope and applications. We sought in our qualitative studies to explore and identify several key sources of wellbeing for Māori individuals. Nine interviews were conducted with members of Māori communities to identify key themes of Māori wellbeing. We performed a Reflexive Thematic Analysis on these data and then conducted a further fifteen interviews to revise, refine, and reposition the previously generated themes. The Ngaruroro model describes wellbeing as the embodied and active process of being well in relation with one’s (1) here tāngata (social and familial ties), (2) te taiao (the environment), and (3) taonga tuku iho (cultural treasures) while doing what one can to make lifestyle choices that are conducive to the health of one’s (4) tinana (body) and (5) wairua (spirit) while cultivating a balanced (6) ngākau (inner-system), fulfilling (7) matea (core needs) and exercising your (8) mana (authority). These themes illustrate that Māori wellbeing is dynamic, interconnected, and holistic.

## 1. Introduction

Indigenous peoples around the world are striving to break free from the enduring legacy of violent Western European colonial practices, institutions, structures, processes, and systems that afflict our communities [1,2,3,4,5]. Our collective struggles for liberation and self-determination seek truth, justice, and better worlds for those who are yet to come [5,6]. Critical to this endeavour is the wellbeing of our people, which is intertwined with the flourishing of our ancestral knowledge systems, lands, waters, and other non-human relatives [7,8]. The arrival of the British settlers in Aotearoa (New Zealand) in the late 1700s, the foreign diseases they introduced, the settler colonial institutions and systems they established, land theft, and other forms of calculated colonial violence inter-generationally crippled the wellbeing of Māori—the Indigenous people of Aotearoa [9,10]. This incursion led to Māori populations plummeting and the destruction of Māori social, economic, environmental, health, legal, and knowledge systems [1,2,4,9,10].

Aotearoa has two constitutional documents He Whakaputunga o Te Rangatiratanga o Nu Tireni (The Declaration of the Independence of New Zealand, 1835) and Te Tiriti o Waitangi (1840). The realisation and honouring of these legal documents are central to the collective flourishing and wellbeing of Māori peoples [11,12]. These documents affirm the rights of Māori to sovereignty, self-determination, authority over lands, natural resources, and other cultural treasures that existed long before the arrival of British settlers [1,13].

### 1.1. Foundational Literature—Ngā Rangatira

Following the devastating impacts of the 18th and 19th century colonial period, the latter half of the 20th century saw significant Māori cultural revitalisation in Aotearoa. Wayfinding Māori leaders (re)built institutions, revived te reo Māori (Māori language) and tikanga Māori (Māori protocols and customs), and sparked many important social and political movements like the Māori Women’s Welfare League, Kōhanga Reo (Māori immersion pre-schools), Kura Kaupapa Māori (Māori immersion primary schools), Whare Wānanga (Māori institutions of higher learning), and the Māori Language Act [14]. This ‘Māori renaissance’ period emerged alongside ground-breaking work by Māori scholars like Winiata [15], Jackson [1,13], Mead [16,17], Walker [10], Smith [18], Durie [9,12], Mikaere [19], Smith [2], Marsden [20], and Pere [21], whose collective works initiated institutional reform across many fields of research and policy, including economics, law, health, education, arts, resource management, and the Māori language. Consistent across these works is a strong belief in the capability of Māori communities to be self-determining and in their ability to design and deliver the systems and solutions to achieve wellness for Māori communities. Their achievements through the struggle for decolonisation, liberation, and tino rangatiratanga (self-determination) have established prime conditions to ensure the success of ongoing work to reindigenise, and thereby restore, the wellbeing of Māori peoples.

### 1.2. Landmark Māori Wellbeing Models—Ngā Pou

Over the past 40 years, Māori scholars have introduced models and frameworks of health and wellbeing with varying scopes and purposes. Two models have had a considerable influence on Māori development: Te Wheke [21] and Te Whare Tapa Whā [9]. These models champion the holistic and culturally specific needs of Māori in relation to health and wellbeing. Te Wheke and Te Whare Tapa Whā revolutionised the way Māori health and wellbeing are understood and have been foundational to Māori health promotion, practice, education, and research. These and other Māori health and wellbeing models vary in their degrees of simplicity vs. complexity and cultural accessibility vs. cultural inaccessibility, which then impacts their applicability to differing contexts. Te Whare Tapa Whā is concise and accessible, which has seen the model broadly applied across various contexts. It has become a key point of reference for holistic understandings of wellbeing for both Māori and non-Māori in Aotearoa. Te Wheke, on the other hand, is a strong model in how it incorporates more traditional and complex Māori philosophical concepts like hā (breath of life), mauri (lifeforce), and mana (authority). The incorporation of these cultural concepts means a deeper level of Māori cultural understandings is required than for the Te Whare Tapa Whā model. McNeil [22] observed how using complex Māori concepts can make models harder to use, especially for Māori who have not had the opportunity to be exposed to such cultural learnings.

Over 40 years since the inception of Te Wheke [21] and Te Whare Tapa Whā [9], our Māori worlds have changed dramatically and have become increasingly complex and diverse. It is therefore crucial that Māori have a range of wellbeing models and frameworks that can be applied in different contexts for different issues in te ao hurihuri (the contemporary world) that Māori face. The challenge is to create models that retain the simplicity of Te Whare Tapa Whā, uphold the integrity of Māori cultural concepts like Te Wheke, and account for the impacts of colonisation and recent reindigenisation that shape contemporary Māori existences [22].

### 1.3. Recent Developments in the Māori Wellbeing Literature—Ngā Hua

Although Te Wheke [12] and Te Whare Tapa Whā [9] are the most widely known and used, a range of other Māori health- and wellbeing-related models have been developed over recent years. Wilson and colleagues [23] systematically reviewed the literature pertaining to Māori health and wellbeing models to inform the development of a Māori-centred relational model of care. In their review, the authors detailed and described the Hui Process [24], the Kapakapa Manawa Framework [25], The Meihana Model [26], Te Hā o Whānau [27], Te Kapunga Putohe [28], Te Punga Oranga [29], Te Whare Tapa Whā, Te Wheke, and Te Whetū [30]. The narrative overview of the article’s first theme, ‘dimensions of health and wellbeing’, is relevant to the present research as it provides valuable insights as to how Māori wellbeing is conceptualised and understood across these various models [23]. Wilson and colleagues identified four key dimensions of wellbeing: wairua (spirit), whānau (extended family network), hinengaro (the mind), and tinana (physical). These observations are consistent with other authoritative pieces of Māori scholarship that speak to Māori understandings of wellbeing and worldviews more broadly [9,20,21].

Pae Mahutonga [31], He Ara Waiora [32], Atua Matua [33], and Whiti Te Rā [34] are notable Māori health- and wellbeing-related frameworks that were not included in the review by Wilson and colleagues [23]. These four frameworks also present holistic, inter-related, and interconnected dimensions of Māori wellbeing. Further, they are useful in illustrating how different wellbeing frameworks have their own unique backgrounds, functions, and strengths. He Ara Waiora, for example, was developed to support the New Zealand Treasury in incorporating te ao Māori (Māori world) perspectives into policy relating to tax and living standards and government initiatives and investments. Whiti Te Rā was designed to help whaiora (Māori health clients) explore their level of knowledge, comfort, and active engagement with Māori pathways to wellbeing.

The research endeavour described in this article is part of a new era of Māori scholarship that seeks to model and psychometrically measure Māori cultural capacities, concepts, connections, and identities related to wellbeing [35,36,37,38,39,40,41,42]. This surge of Māori quantitative research reflects a growing appetite for measurement tools that are grounded in both te ao Māori and quantitative scientific paradigms.

Modelling and psychometrically measuring Māori wellbeing is a nuanced and multi-faceted endeavour. Durie emphasises the importance of understanding and measuring wellbeing across individual, whānau, and population levels [43]. He posits that there can be no single measure of wellbeing. This perspective is crucial in model development contexts, as individual and whānau levels of wellbeing are inherently distinct and necessitate tailored approaches and methodologies. While the previously discussed models each have merits and serve their own intended purposes, there remains a gap in the literature for a model grounded in the lived experiences of Māori people and their self-identified sources of wellbeing. Additionally, there is a need for flexible models that can be applied across both individuals and groups to measure Māori wellbeing effectively.

### 1.4. The Goals of the Current Study—Ngā Whāinga

The primary goal of this study is to fill the gap in the literature for a model of Māori wellbeing that is directly grounded in the lived experiences of Māori people and that is specifically designed to identify sources of wellbeing for Māori individuals. To achieve this goal, themes of wellbeing were discerned from interview responses obtained from everyday Māori people. A second set of interviews with different community members further explored and refined the themes from the first set of interviews. These revised themes were unified and formalised within a new model of Māori wellbeing presented here.

A second goal of this paper is to highlight the many existing Māori health- and wellbeing-related frameworks. We assert that the development of new models and frameworks should be about addition not competition. This distinction is crucial as some argue that the introduction of new models suggests existing ones are incomplete, inadequate, or insufficient. We contend that various models and frameworks are tools to be wielded by different people, in a variety of contexts, to create positive change in our communities.

## 2. Materials and Methods

### 2.1. Broader Research Project

This present qualitative study is part of a larger research project which aims to quantify wellbeing in the Māori community at the individual level. The approach described below was employed to identify a reasonably small number of wellbeing themes (namely between 5 and 10) and a handful of corresponding items (between 4 and 7).

### 2.2. Research Approach

This research was conducted from an emic, cultural insider perspective that utilised an Indigenous culture in psychology approach [44], specifically, using a Kaupapa Māori methodology. Kaupapa Māori is a tool of praxis designed to support scholars in taking action to resist Western epistemic hegemony and to conduct transformative research for the betterment of Māori communities [18]. This approach involves critiquing and resisting dominant Western forms of knowledge production, dissemination, and privileging, while also normalising and prioritising Māori worldviews, practices, knowledges, and principles [2]. The Kaupapa Māori methodology was built into this research by engaging in embodied and embedded relational practices that were enacted and guided by tikanga Māori (Māori customs) and grounded in Māori values.

An example of this approach was FJ travelling to the house of a participant who is an elder. He arrived in the morning, talked about life, and shared stories over cups of tea. He then held the interview, continued conversations while eating, and then left later in the afternoon. In this process, the interview was grounded in whakawhanaungatanga (relationship building and strengthening), manaakitanga (hospitality), and aroha (love; affection).

An intersubjective approach to data analysis was another way we employed Kaupapa Māori. Reflexive Thematic Analysis (RTA) typically focuses on individual-level subjectivity, reflexivity, and meaning-making [45,46]. Our research instead focused on shared meanings and collaborative sense-making. We prioritised collective interpretations and understandings by returning to the participants after an initial analysis of their data to confirm that their ideas were adequately reflected in the findings. We then sense-checked with a colleague during the coding process and then with a separate advisory rōpū (group) during the theme-generating process. Collective sense-making also occurred in the second set of interviews (with different participants) to further refine and validate the model.

Together, this emic Kaupapa Māori methodological approach enabled the research to be more authentic, culturally responsive, enjoyable, ethical, robust, and safe.

### 2.3. Advisory Rōpū—Ngā Kaitiaki

A Māori advisory rōpū consisting of academics, community leaders, and traditional knowledge holders was established to support the wider research project wherein the present research sits (see Table 1). This collective provides critical additional layers of support and cultural security through the integration of diverse perspectives and decision-making guidance. Their ongoing guidance helps protect the integrity of our research project by ensuring the researchers do not engage in unsafe research practices and thus protects the Māori community from unforeseen negative implications. Meetings are convened when critical issues arise, key findings emerge, or during significant transition points in the research. Members were also engaged throughout the analysis. The critical input of this group is acknowledged in the collective authorship of this paper.

### 2.4. Participants in the Qualitative Interviews

Nine individuals participated in the first set of interviews (see Table 2) and fifteen in the second set (see Table 3). Both sets of participants constituted a diverse sample of Māori who varied among the demographics of age, gender, iwi (tribe), te reo Māori (Māori language) proficiency, cultural embeddedness, and tertiary education experience. These participants were carefully selected by FJ through personal networks to ensure the sample captured a diverse range of Māori voices. All participants were given the opportunity to be anonymous in this report, but all provided informed consent to participate in this research and to have their name and iwi affiliations listed. Participants were given the option to be identified so that they could exercise their right to proudly own their quotes. This provision also allows for participants’ quotes to be contextualised by their positionality, experience, and expertise.

### 2.5. Data Collection

The Victoria University of Wellington Human Ethics Committee granted ethical approval to conduct both the first (ID: 00000028603) and second (ID: 00000029558) sets of interviews.

#### 2.5.1. First Set of Interviews

Three of the interviews were conducted online and six were conducted face-to-face. Before the interviews commenced, time was set aside for whakawhanaungatanga (relationship building). Then, face-to-face participants were given the necessary consent form and reminded of their rights as a research participant. Online participants were emailed the consent form in advance. The interviews then began with karakia (incantation) either by the researcher or the participant. Following the karakia, recording commenced. These interviews were largely guided by the participant’s kōrero (flow of discussion). Once the participant had exhausted the list of questions on the question sheet or had expressed that they were satisfied with their responses, the recording was stopped, the interview was concluded with a closing karakia, and kai (food) was shared. Upon completion, participants were provided with a small koha (offering in the form of a supermarket voucher) as a token of appreciation of their contributions.

#### 2.5.2. Second Set of Interviews

All fifteen interviews were conducted online. All the same protocols and processes relating to whakawhanaungatanga, consent, and opening the interviews were the same as the first set of interviews. Then, participants in this second set of interviews were first tasked with listing things that were important to their wellbeing (e.g., going for bush walks or spending time with grandkids). Their list was then sorted into groups of related items (e.g., grouping food, sports, and sleep together). The interviewer then presented the themes that were identified in the first set of interviews and invited the participant to map their list of groupings onto the different themes. When an item did not map onto one of the existing themes, it was noted, and the reasoning was explored. Finally, participants were asked to provide feedback on the model such as “what do you think of this model of Māori wellbeing?”, “how could this model be improved upon?”, and “would you add or remove any themes or items?”.

### 2.6. Data Analysis

#### 2.6.1. First Set of Interviews

A Reflexive Thematic Analysis (RTA) was performed on the collection of transcribed discourse from the first set of interviews. The RTA generally followed the guidelines outlined by Braun and Clarke [45,46] but was adapted to fit Kaupapa Māori philosophies, principles, and priorities [2,18]. Firstly, the level of analysis was identified. We transcribed the interviews clean verbatim and only analysed the manifest content (rather than recording and analysing the more nuanced details like pauses, body language, reactions, and silence). To begin the analysis, FJ manually transcribed the interviews, which helped to foster familiarity with the data. The transcripts were then sent back to the participants to give them an opportunity to add, remove, or update their transcript. After receiving the transcripts back, FJ re-familiarised himself with the transcripts and began coding the data.

The first step in the analysis involved making notes when particular points, ideas, phrases, or concepts were expressed. The transcripts were then opened with the qualitative coding programme NVIVO, and initial nodes were created based on the note taking process. New nodes and sub-nodes were generated as the transcripts were coded. Support from TR was enlisted during the coding process. A collaborative approach was taken that involved ‘sense-checking’ (cross-referencing codes and relevant cultural concepts) and exploring different interpretations of the data that ultimately enriched the analytic process. This process supported the identification of patterns, and key themes, which resulted in a further refinement of the taxonomy.

Themes were organised into au (streams) of wellbeing, and TR was approached again to discuss the links between the items, themes, and the corresponding cultural concepts. The resultant codes and themes were discussed with PJ and TN to ensure the analysis remained grounded in intersubjective understandings. Following this step, FJ engaged in a ‘member reflection’ process [47], whereby participants were provided with one-page summaries of their three most prominent themes, each with supporting quotes. Thus, participants were given an opportunity to determine whether their ideas were being fully captured, to provide additional insights, and exercise autonomy over how their experiences were being articulated. Lastly, the themes and items were presented to the advisory group and were the subject of collective wānanga (group consideration and deliberation), which resulted in various conceptual and linguistic tweaks being made and the formation of the tentative model.

#### 2.6.2. Second Set of Interviews

During the second set of interviews, participants were given the free-listing and sorting tasks and were asked a range of questions in response to the draft model. After all the interviews were completed, participants’ responses were compiled into a master document, which allowed us to visualise their responses all together. We then created a separate document that further organised the responses. This process resulted in a streamlined list of feedback on the model with suggested changes. The suggestions were systematically assessed by FJ, and this document was then revised by PJ, TN, and PW, who collectively reviewed the suggested changes. After the group review, FJ revisited all the qualitative data and made another set of revisions to the model.

The revised themes and their corresponding items were presented back to the advisory group for further sense-checking, conceptual clarification, and testing of theoretical coherence. This collective process resulted in minor reconceptualisations and adjustments that greatly improved the coherence of the model.

Through our RTA, we generated a Māori wellbeing model consisting of eight themes, encompassing thirty-one items. The collective wānanga processes saw the initial model revised and re-worked into eight themes encompassing thirty-six items. The last of the analyses confirmed the final model consisting of eight themes encompassing forty-one items. Below, we explain and unpack these themes, provide supporting excerpts, and list the corresponding items.

## 3. Results

### 3.1. Here Tāngata

**Figure 1:**
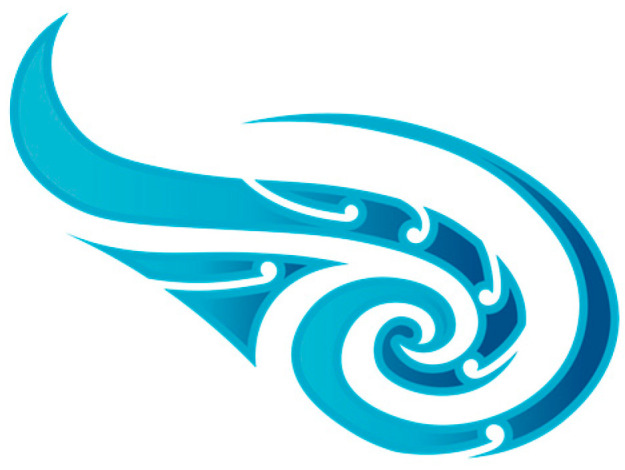
Theme icon for Here Tāngata.

This first theme is about social and familial relationships. The name ‘Here Tāngata’ refers to the ties that ground us and keep us connected to one another (Figure 1; Table 4). Participants emphasised the importance of being connected to and living in a good relationship with other people for wellbeing. Participants described the importance of being connected to whānau members (family), their hoa (friends), members of their hapū (sub-tribe), others with the same iwi (tribal) affiliations, members of their different hapori (community), and their tīpuna (ancestors). Developing and maintaining these connections was said to deeply nurture one’s wellbeing. Out of all these social and familial ties, the connection to whānau was emphasised the most. Participants described their wellbeing as being intrinsically linked to the wellbeing of their whānau members. If an individual whānau member experiences difficulties, it “affects the whole whānau” (Stevie).

*“Well I mentioned about whānau and that is family. These relationships are a very important part, they affect us deeply, our whole well-being. Their living standards, the way we live together, the things that they do that affects us, what they do well you rejoice, when they don’t do well or when they are unwell and make mistakes you are saddened”—Kahu*

Consistent with Māori cultural beliefs related to whakapapa (genealogy), participants described the importance of being and feeling connected to their tīpuna. There was a strong consensus among the participants that their ancestors play a significant role in who they are, what they do, and where they are going in life.

*“Knowing who my tūpuna were, knowing what they did and achieved in their lifetimes and the attributes they had means that, for me, I suppose that the experiences I learnt from my tūpuna absolutely explain to me what I was doing, why I was doing it, and where I have seemed to have gotten. Some of the natural abilities and skills that I have are passed down, that’s what my tūpuna used to do. So absolutely, major. That connection, and the understanding that these weren’t just skills I had learnt through going on courses, they were already embedded within me, which means you know, the more you know about your tūpuna the more you can choose to actively grow in a particular area or not. Yeah, so apart from you know, our tūpuna being there for us, if we want to access that guidance and strength we can.”—Denis*

Another aspect of social and familial ties that participants greatly emphasised relates to the connection to hapori. Throughout the interviews, examples of hapori included a variety of groups including sports clubs like “Morehu Māori basketball” (Monica), “your neighbours” (Stevie), and “local service providers” (Kahu). Many participants described engagement and active participation in hapori as key for their wellbeing.

*“Definitely like support from my community, like I feel so frustrated, like so hōhā if I’m not going to my meetings. Like I go to 12 step meetings and that’s so important for me like I just need connection with other people with the same experiences and understandings as me.”—Victoria*

### 3.2. Tinana

**Figure 2:**
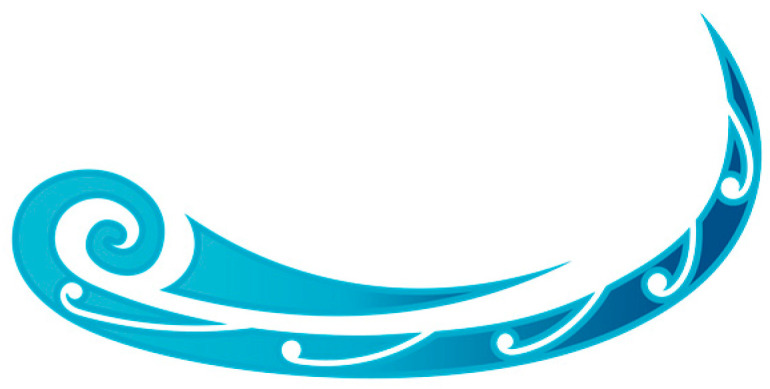
Theme icon for Tinana.

This theme is about living well in relation to your tinana (body) and maintaining physical health. Doing what you can to make healthy lifestyle choices related to your tinana was identified as foundational to wellbeing across the interviews (Figure 2; Table 5). Participants described the importance of eating nourishing kai (food), engaging in some form of kori tinana (physical activity), having good-quality and sufficient moe (sleep), having kanohi kitea (your face seen) by being physically present in spaces with others, and mitigating harms related to kai whakapiri (substances like alcohol, marijuana, and methamphetamine that some people use to feel a sense of connection and/or self-medicate). In addition to going to the gym and playing sports, participants highlighted the importance of participating in culturally relevant activities, games, and practices like mahi ruku kai (diving for seafood), kī-o-rahi (a traditional Māori ball game), kapa haka (Māori performing arts), and mau rākau (a Māori weaponry art).

*“Exercise is really important to me, and I make a real effort sometimes to do my dose of exercise but afterwards I’m really glad that I did and I think I probably wouldn’t have been as healthy as I am if I had not been exercising most of my life”—Clive*

Being physically present at events and having your face seen is of cultural significance to Māori on a number of levels. It can foster whanaungatanga (interpersonal connection), show commitment to kaupapa (initiatives), and support the more embodied and experiential-based spiritual practices. These interviews were conducted not long after the New Zealand COVID-19 lockdowns, which highlighted the importance of being together with others face-to-face and how videocalls could not provide the same experience.

*“Physical presence is an important part because you can feel other people’s wairua as well, and as humans we need that. We need to be able to touch, smell, see, sense, grab. I know that’s an important part for me.”—Stevie*

A point of emphasis across the interviews was that physical wellbeing is often about “doing the basic things (that we all know we should do) properly” (Te Matahiapo). Sleep was highlighted as one of the areas of priority as it greatly impacts other domains of wellbeing.

*“A lot of it is like really basic stuff, like sleep is number one, have to get enough sleep.”—Victoria*

### 3.3. Ngākau

**Figure 3:**
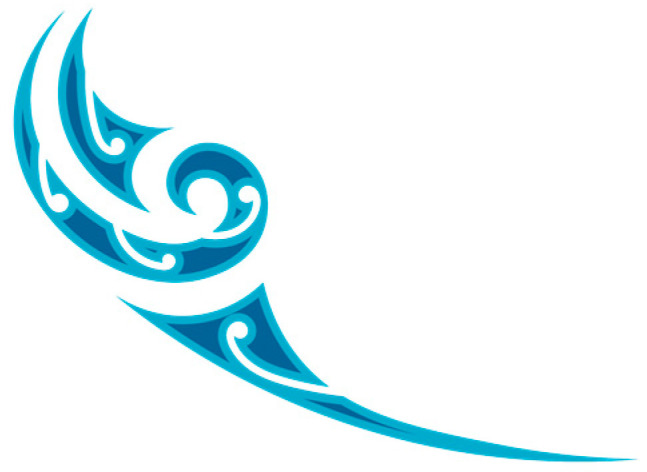
Theme icon for Ngākau.

This theme is about psychological and emotional capacities that contribute to a balanced ngākau (heart; seat of affections; internal system). The most prominent of these items among participants related to kare-ā-roto (emotions), whakaaro (thoughts), waiaro (attitudes), aroha (love), and pāmamae (trauma, grief, deep pain; Figure 3; Table 6). We drew upon the whakapapa kōrero (Māori ancestral teachings) relating to traditional ways of knowing provided by Smith [48] in naming this theme. These understandings relate to the decentralisation of thinking from the brain to other parts of the body. The ngākau was selected to conceptually couch this theme over the more commonly used concept hinengaro (mind) as Smith provides examples of whakapapa kōrero that suggest the ngākau is responsible for the repository of rational thought, embodied knowledge, emotions, feelings, and memories, while responses centred in the hinengaro and roro (brain) are perceived as more fleeting or impulsive.

Participants described the importance of being aware of, monitoring, and addressing one’s whakaaro. This orientation was said to be important for wellbeing as, if left unaddressed, negative thoughts can spiral out of control, leading to views being exaggerated and blown out of proportion, affecting your relationships (Kahu).

*“Controlling your mind is an ongoing thing and it is a difficult thing. The mind is difficult to control but that’s where all of our thoughts come from. It’s from our mind and we have to strive to think positive thoughts and when we think negative, we have got to work on it, get rid of it, talk to our mind, we gotta control our mind.”—Kahu*

The ability to experience, process, and work through different emotions was highlighted by several people as important to wellbeing. Participants spoke on the importance of not trying to control your emotions but instead being able to experience and navigate them in a healthy way (Victoria).

*“When I start feeling a bit resentful about something or a bit put upon or ungrateful, that’s real bad because then everything will kinda go out the window so I just have to be really careful about you know like getting angry and stuff like that because it’s no good.”—Victoria*

Participants highlighted the power and influence of having positive waiaro on wellbeing. This approach included the ability to approach situations with positive and challenge-oriented mindsets and maintain “an overall optimistic outlook in life” (Monica). It also included understanding what control you do and do not have about your wellbeing. For example, Te Matahiapo described how “you can’t control the weather, but you can control whether you do a karakia or not”. Similarly, others spoke to the importance of being intentional with how and where you place your focus and energy.

*“You can either choose to give into the hopelessness that the world can kinda exert on us or you can choose to look at it in other ways. This is going to sound cheesy, but focus on the positive instead of the negative, which is what I do. If I see a situation that I’m like man that’s no good, and I start to feel that its impacting my waiora, I start to think about what I can do to help or what isn’t in my control and then I’m able to pack it away and let it go and move on”—Stevie*

### 3.4. Wairua

**Figure 4:**
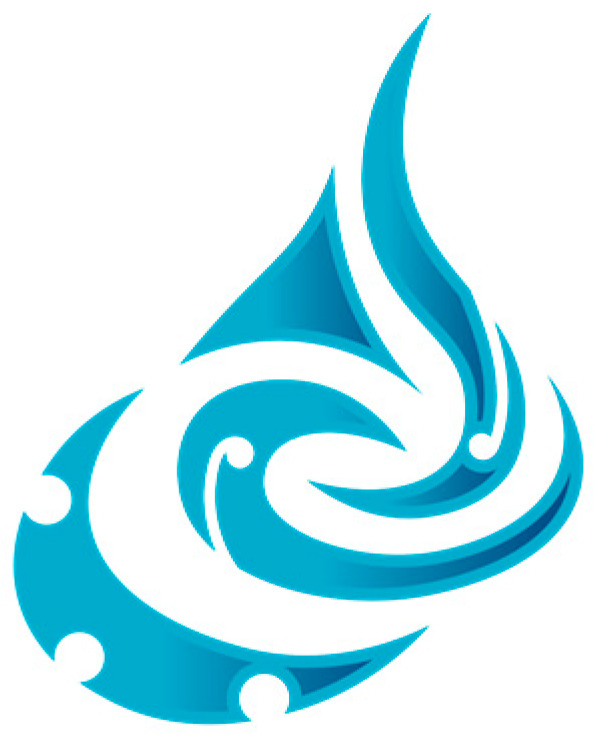
Theme icon for Wairua.

This theme is about living well in relation to wairua (spirit, interconnectedness). Doing what you can to make healthy lifestyle choices related to wairua was a paramount concern for the wellbeing of participants (Figure 4; Table 7). Wairua is often defined, interpreted, and experienced differently from person to person. Tau, a wairua practitioner, illustrated this point concisely when he said “If you asked 100 people about what wairua is, you’ll get 100 different answers”. It is important to note that when many participants talked about wairua, their descriptions extended beyond the typical Western notions of religion and spirituality. This included embodied experiences and relational practices. Participants described wairua as being important for their wellbeing through the connection to atua (Māori deities; ancestors with continuing influence), the closely related experiences of wana (exhilarating and breath-taking experiences) in response to displays of ihi (essential lifeforce/personal magnetism) and feeling wehi (the feeling of awe or fear in response to ihi), being in wāhi wairua (spaces that nurture your sense of wairua), engaging in mahi aroha (activities or work that they do out of love, passion, or service), and living in a way that is aligned to ‘poipoi i te mauri’ (nurturing the lifeforce of the beings, spaces, and things around you).

Some participants discussed the importance of spiritual sustenance and the nurturing of wairua from a religious point of view for wellbeing. Kahu is a staunch believer in the power of prayer and sees her strong wairua as the backbone of her wellbeing; “I still think my wairua is the strong point, it always has been through my life”. This notion of wairua being the foundation for wellbeing was echoed by other participants who described a fed wairua being just as or even more important than a fed stomach.

*“I know growing up in my family, a large family of 18, that our wairua was fed. So although we didn’t have a flash home and stuff, we were always inspired. You know if your wairua is inspired, if your wairua is fed good kai, good nourishment of the mind and of the soul, we can achieve anything.”—Tau*

Mary described the key role of wairua in connecting us to people, places, and everything around us; “I think in my personal circumstance there is the wairua aspect that is really critical for me … It’s the portion for me that helps me connect in a different way to everything around me”. Te Matahiapo touched on this role of wairua in connecting us to our surroundings while speaking to the link between atua and te taiao; “the Māori belief system looked at our taiao or our land as our deities so you know we can touch our deities, we can touch our gods, we personify the environment and they become our deities”. This notion of te taiao and atua being a source of spiritual nourishment was salient across the participants.

*“Absolutely key, you know we are spiritual beings having a physical experience really, and the whole aspect of wairua in our world* *,* *is with us every day… So we have a mindset, a way of looking at the world that is personified by our atua, when we look around us we see Tāne, we feel Tāwhiri-mātea, we feel Tama-nui-te-rā, so that’s a different way of thinking and framing the world. So wairua is absolutely key to waiora”.—Denis*

The experiential and embodied aspect of wairua beyond engaging with te taiao was highlighted across interviews with participants describing experiences of wana (breath-taking or exhilarating moments) as spiritual experiences.

*“I think that deep down, not necessarily in a religious way but I think everyone gets that feeling you know like I don’t know maybe it’s just like going into the country for the first time and it gets dark and then you just see more stars than you ever thought possible and it just hits you… like I think everyone feels that kinda spiritual thing but maybe they just don’t know what it’s called”—Victoria*

### 3.5. Taiao

**Figure 5:**
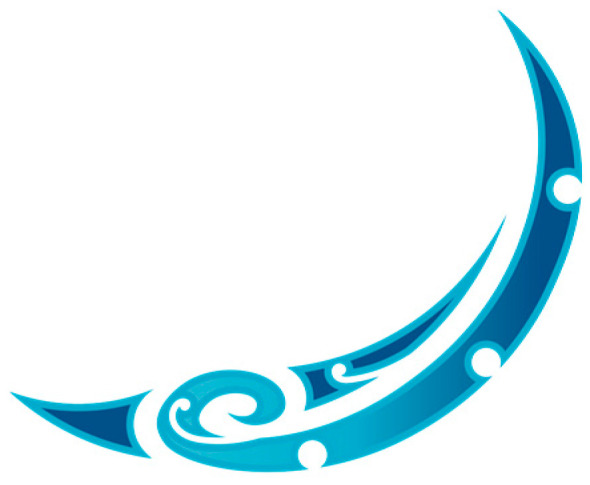
Theme icon for Taiao.

This theme is about connections to te taiao (the natural environment). Participants talked about the nourishment they obtain from being connected to the various domains of te taiao including whenua (land), wai tai (bodies of salt water), wai māori (bodies of fresh water), ngahere (bush and forests), and ngā rangi (celestial bodies; Figure 5; Table 8). In the interviews, it was made clear that we are inseparable from te taiao and that our wellbeing is dependent on many aspects of our natural environment. Denis made this clear when he said “we can’t be well if Papatūānuku is not well”, where Papatūānuku refers to the Earth Mother. Participants talked about the importance of drawing upon Māori belief systems in their entirety when exploring a concept from a Māori perspective. Te Matahiapo highlighted how a whakapapa (Māori cosmological genealogy) approach to wellbeing links our wellbeing to all aspects of te taiao as they are our tīpuna, our family.

*“Oranga is not just tied into te ora o te tangata, ko te oranga o te tangata, te taiao, te whenua, o ngā wai, o te rangi, all of it, is all encompassing”—Te Matahiapo*

The active and embodied process of being engaged with and embedded in te taiao was a key source of wellbeing for many of the participants. Going for walks in the ngahere, swims in the moana (ocean) and awa (rivers), hikes up maunga (mountains), and observing and aligning oneself to the marama (moon) and whetū (stars) were all explained to be grounding practices, conducive to improving wellbeing.

*“For me, having my hands and feet in the ground or in the earth and you know linking with the energy of Papatūānuku is an important aspect, as is you know swimming in the sea and the rivers, as is venturing into the ngahere, those different domains of our atua and the different energies that come from those domains”—Denis*

Participants explained that various aspects of te taiao held various levels of significance to them based on their ancestral connections. For some people from more coastal iwi, connection to the moana was more important, and for some people from more inland iwi, connection to the ngahere was more important. Another factor related to the significance of these place-based connections was whether the participants had whakapapa connections to the feature of te taiao. For example, some participants felt an extra special type of replenishment and nourishment from being on their ancestral maunga. Stevie described how although it was not the same, she would climb local maunga and seek out high points that resemble being on her own maunga.

*“Whitireia isn’t my tūrangawaewae as such but I love going up there and just sitting there and it feels so good. Like it almost feels just as good as being on my tūrangawaewae in Tauranga, Mauao”.—Stevie*

The intimate connection between the participants and te taiao was described as deeply spiritual by some and an inseparable bond by others.

*“you cannot disengage or just disconnect yourself from the whenua just like you can’t disengage yourself from the moana. We have such strong connections to our place and I think those are important”.—Monica*

### 3.6. Matea

**Figure 6:**
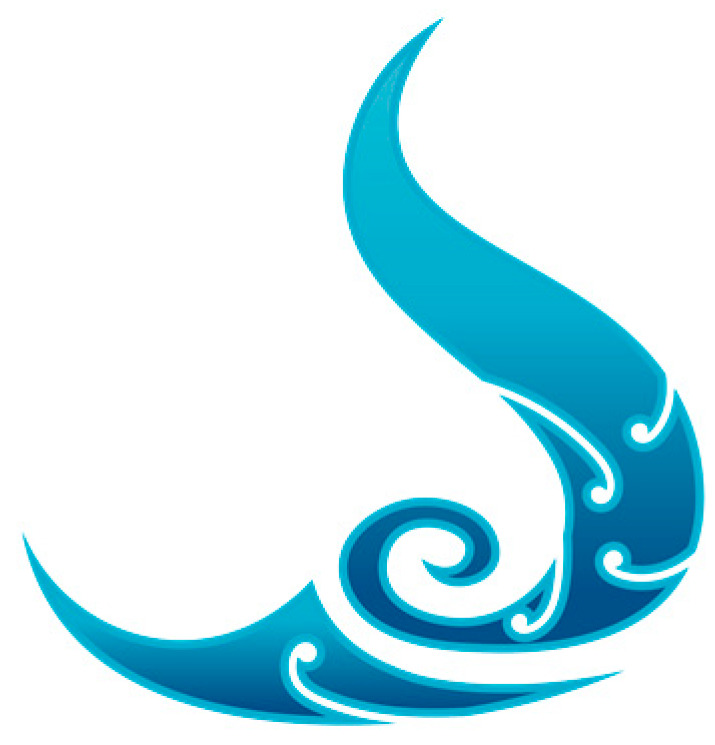
Theme icon for Matea.

This theme is about capacities to meet core matea (needs). These needs include whai mātauranga (acquiring knowledge), tuku mātauranga (passing on knowledge), kainga (housing), pūtea (money), and wā whakatā (relaxation). This theme recognises the very real impact of social and economic systems on wellbeing (Figure 6; Table 9). Many participants spoke of the struggles associated with poverty and low socio-economic status and how it directly impacts wellbeing.

*“you know you can’t be in the state of ora if you don’t have a decent house, decent job, decent food, decent clothes, you know these all contribute in one way or another to whether we’re in a state of ora”—Te Matahiapo*

*“Another thing that really is really important for my wellbeing is living in a beautiful comfortable location and living in a house that provides shelter and warmth.”—Clive*

Knowledge transmission was found to be an important source of wellbeing and included aspects of both whai mātauranga and tuku mātauranga. Participants discussed the importance of acquiring different forms of knowledge from both te ao Māori and te ao hou (contemporary world) for navigating their academic, work, social, and personal lives. Similarly, participants discussed the role of transmitting mātauranga to whānau, friends, and other community members for their wellbeing. This striving included teaching others in both formal and informal contexts.

*“as a whānau we’re always striving to learn, most of us would have to have something to do and what I’m enjoying at the moment the fun that I’m having and the excitement that I get from teaching my mokopuna and just singing you know just yeah rhymes and that kind of stuff it’s yeah so that’s actually bigger than we think”—Mary*

*“actually being able to share our experiences with each other not just as siblings but all our nephews and nieces so everything we’ve learned in experience it’s about sharing that knowledge sharing the growth so that our our babies can flourish in a different way”—Mary*

### 3.7. Mana

**Figure 7:**
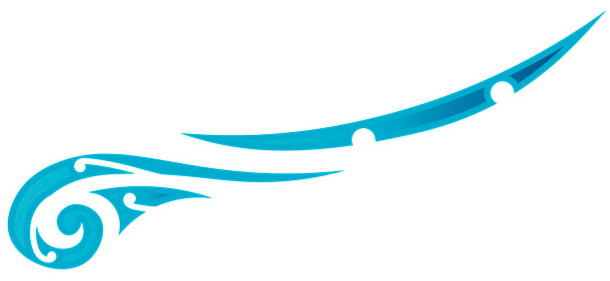
Theme icon for Mana.

This theme is about capacities related to exercising mana (authority). These capacities include to tū tangata (express and stand in the fullness of your identity), whiriwhiri (make key decisions about how your life unfolds), manaaki (uplift, take care of, and/or be hospitable to others), whakatere (navigate challenges in life), and tū toa (stand accomplished in a skill or area; Figure 7; Table 10). The importance of knowing who you are and the capacity to stand tall in your identity (including intersecting identities related to gender, sexuality, and religion) was a strong theme across the interviews.

*“So I think that if you don’t have a solid identity, we can be really influenced by whatever comes across our paths. If we have a solid identity and we are really clear about who we are, while we might engage in all of those things, having a solid identity means we filter it through who we are, rather than seeking for identity in what’s in the next guru or the next movement that comes along”—Denis*

*“I think the most important aspect would be having a good sense of who you are, of understanding where you fit into the community, understanding where you fit in your whānau, understanding where you fit in your network of friends and acquaintances and that you feel confident to contribute in a way that is positive and enhancing”—Clive*

Of the various identities, most participants discussed the paramount importance of knowing who they are in terms of their Māoritanga (Māoriness) and its contribution to their wellbeing.

*“I think being Māori what it does for me is gives me an understanding of our cosmology, you know it does because that gives me the riches of who I am and that is what I take into my world”—Tau*

### 3.8. Taonga Tuku Iho

**Figure 8:**
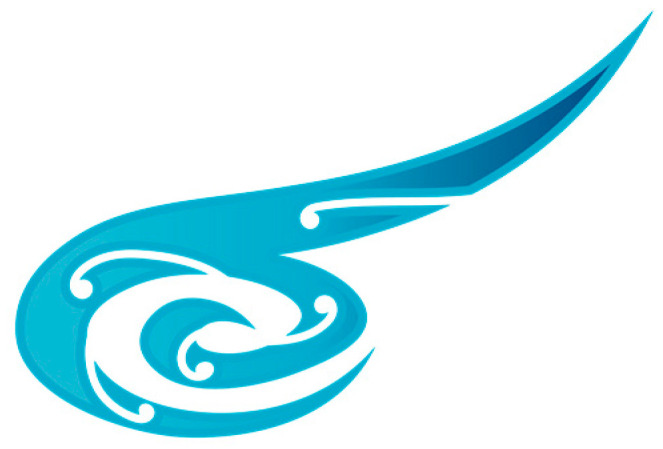
Theme icon for Taonga Tuku Iho.

This theme is about connections to taonga tuku iho (cultural treasures that have been passed down through generations). Taonga tuku iho often broadly refers to things that have been passed down through generations. In this theme, it refers to cultural treasures that sustain wellbeing, including te reo Māori (the Māori language), mātauranga Māori (traditional and contemporary Māori knowledge), tikanga Māori (Māori customs, protocols, and ways of being), uaratanga Māori (Māori values), and tūrangawaewae (traditional and contemporary places of belonging; Figure 8; Table 11). Multiple participants identified wharenui (meeting houses) and urupā (burial grounds; cemeteries) as traditional places of belonging where they go to feel connected to their tūrangawaewae. Other participants talked about the significance of certain areas, neighbourhoods, and family houses.

*“That is where I go back to, to replenish. With all of my tūpuna, my parents now, aunties, uncles, cuzzies they are all in our urupā you know that’s where all that connection is, and that’s the first place any of us go when we arrive at our marae. That’s where we go, to our urupā and then come back to the whare and catch up with the cousins and that. So yeah absolutely crucial”—Denis*

The connection to te reo Māori through speaking, listening, and understanding the language was described by many to be a key source of wellbeing.

*“I think that without that reo I constantly just constantly have the gap, this hole in my heart I guess. That’s until I get that reo, I’ll be confident to fully engage in kōrero Māori. I’ll get a better sense of my identity and a better sense of wellbeing*. *I don’t know, I mean I could learn te reo Māori and still feel the same as I did before but right now it’s definitely of significance to my well-being and something I am trying to do everyday”—Stevie*

Participants described the importance of being connected to mātauranga Māori and mātauranga ā-iwi (tribal knowledge). This item includes knowledge relating to history and whakapapa and is linked to aspects like identity as participants explained how holding certain knowledges can help you stand strong in who you are.

*“I can recite my whakapapa back to* *Tahupōtiki* *you know so you can’t tell me that I am not Māori or that I am not Ngāi Tahu because I can tell you exactly how I am”—Victoria*

## 4. Discussion

**Figure 9:**
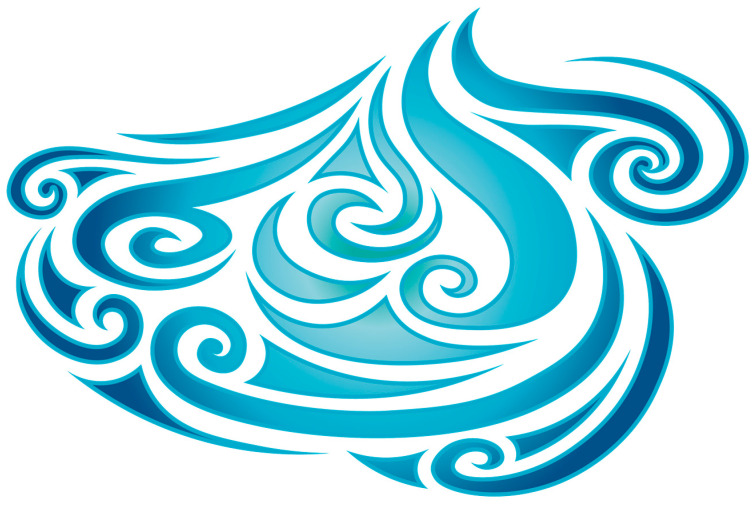
The illustration of the eight themes coming together to form the Ngaruroro model of Māori wellbeing.

This study sought to develop a model of Māori wellbeing that is directly grounded in the visceral experiences of Māori people. The research was specifically designed to identify sources of wellbeing for Māori, so that the resulting model could subsequently serve as a foundation for the development of a self-report Māori wellbeing measure. The first set of interviews generated themes of wellbeing by drawing straight from Māori voices, and the second set of interviews refined them. Together, the resultant eight themes and forty-one items make up the Ngaruroro model of Māori wellbeing (Table 12; Figure 9).

### 4.1. Naming the Model

A respected kaumātua of Ngāti Kahungunu and academic, Dr Joe Te Rito, who is also the uncle of FJ, gifted the model with the name ‘Ngaruroro’ after being presented with the themes and items, as well as the key sentiment that, for Māori, wellbeing is dynamic, interconnected, and flows like streams of water. Ngaruroro is the name of the ancestral awa (river) to which FJ and Dr Joe Te Rito are both connected. Uncle Joe recounted an oral history that was passed down to him by word of mouth from his nanny (FJ’s great grandmother), who he said likely heard it from her father (who frequently gathered food from alongside the Ngaruroro awa). The story chronicles how various parts of the awa were named. The relevant section of the story described the confluence of two tributaries of the river after which FJ’s great grandmother would say ‘tapangia toutia te awa ko Ngaruroro’, i.e., ‘hence the river was given the name Ngaruroro’ [49]. Uncle Joe’s interpretation of this name is that it refers to the turbulence created as the waves from each tributary converged into the main body of the river (ngaru = wave; roro = turbulent). We recognise that there may be other accounts of the naming of the awa, but I (FJ) chose to draw on the oral history of my whānau, passed down through generations.

The name ‘Ngaruroro’ is therefore fitting and significant as it carries the ideas relating to the movement, interconnection, and flow of separate but connected streams while also referencing the ancestral awa that has supported the wellbeing of FJ’s family from Ōmāhu.

### 4.2. Similarities with Previous Research

The eight themes of the Ngaruroro model of Māori wellbeing describe wellbeing as the active process of being well in relation with (1) here tāngata, (2) te taiao, and (3) taonga tuku iho, making lifestyle choices that are conducive to the health of your (4) tinana and (5) wairua while cultivating a balanced ngākau (7), fulfilling matea, and (8) exercising your mana. Many of the themes that have emerged from this research have also been identified in the Māori health- and wellbeing-related frameworks discussed earlier in this paper. Tinana, wairua, taiao, ngākau, and here tāngata will be some of the most familiar as their core concepts map directly onto Te Whare Tapa Whā [9] and very closely to Te Wheke [21]. When looking beyond the main Ngaruroro themes to the items, more similarities can be seen between the Ngaruroro and other existing models. Whiti Te Rā [34], for example, consists of six items, five of which relate directly to themes in the Ngaruroro model: te taiao, wairua, reo Māori, whakapapa, and take pū whānau. The sixth item, mahi-a-toi, is likely to be consistent with the mahi aroha item of the Ngaruroro model. Further similarities can be seen between Ngaruroro and the He Ara Waiora [32] framework. Both models explicitly include domains relating to te taiao, wairua, and Māori values, and the Ira Tangata (human) domain of He Ara Waiora overlaps with items from the here tānagata, mana, and matea themes.

Wairua features in most Māori health and wellbeing frameworks as it sits at the core of Māori ontologies. Our ancestral knowledge systems are predicated on the interconnected co-existence of te ao wairua (spiritual world) and te ao kikokiko (physical world) [20]. In Māori cultural worldviews, te ao wairua is described as the ultimate reality, having the power to impinge on the physical world [20]. Valentine et al. [50] qualitatively explored the many depths and dimensions of wairua through conducting and analysing interviews, and they identified four key themes; (1) wairua is fundamental to human existence, (2) wairua knows no boundaries, (3) wairua is a perceived sensation, and (4) wairua is relational. The Ngaruroro model’s wairua items closely relate to themes three and four as they are both embodied and embedded in relationships. For example, wana is grounded in sensations, and wāhi wairua, mahi aroha, and poipoi i te mauri are about relations with the people, places, spaces, and things around oneself.

### 4.3. Unique Offerings

Despite the many convergences between Ngaruroro and other Māori wellbeing models, a few notable differences can be identified where the Ngaruroro uniquely articulates additional sources of wellbeing. The three most salient offerings are as follows: mana, matea, and taonga tuku iho.

The theme of mana is unique in how it describes relational capacities that are key to understanding one’s sense of agency and efficacy. Items like tū tanagata are important as they make space for the many diverse and intersectional lifeways of Māori who also identify with other cultural, ethnic, faith, or queer communities. It is critical that models acknowledge that Māori identities are linked with, and often shaped by, other intersecting identities. The theme of mana is also unique in how it incorporates items like manaaki, which highlight the link between exercising your own mana to enhance the wellbeing of others and the reciprocal impact on your own mana and wellbeing. Items like this are also important as they articulate dynamics that we often practice as Māori, and that feed into our wellbeing, that we often fail to notice.

The matea theme is another important contribution as it takes into consideration the socio-economic realities that many Māori face as a result of the marginalising settler colonial systems that were brought to Aotearoa [1,2,4,10]. It is important that we acknowledge the impact of these material circumstances on Māori wellbeing and at the same time, see the bigger picture and acknowledge all of the other holistic sources of wellbeing for Māori people. Matea importantly extends the typical notions of ‘core needs’ beyond material resources to also include the gaining and passing on of knowledge, which is consistent with Māori oral traditions as an epistemological pedagogy. Taonga tuku iho is a valuable theme that explicates the utility of our cultural treasures as sources of wellbeing. This idea is not new and is consistent with well-established ideas of ‘culture as a cure’, where connection to ancestral language, knowledge, practices, and ways of being is linked to wellbeing [33,34,35,36,38,40,41]. This view has been researched extensively in the Aotearoa context and has sparked conversations and new lines of research into what it means to be culturally embedded [35,36,37].

### 4.4. Study Strengths

A major strength of this research effort is the unique two-stage qualitative study design. Explicitly testing the themes and items that were generated in the first set of interviews proved highly valuable. This mapping and refinement process greatly enriched the Ngaruroro model overall and provided critical insights into the model’s further development. This advantage was evident in how the model initially contained thirty-one items and ended up with forty-one items.

The diversity of life experiences across the different participants was also a genuine asset to the development of this model. With a total of twenty-four participants, we were able to sample Māori from a range of age groups, genders, iwi, and levels of Māori cultural embeddedness. The diversity of participants was also important to the quality of the study because we wanted to hear from the diverse community, not just from a selection of academics, health professionals, or Māori cultural experts. We believe it is important to acknowledge and listen to the expertise that everyone holds in their own experiences of what brings them wellbeing.

This model’s development was deeply enriched by the practices and processes that were driven by the Kaupapa Māori research approach. Not only was the model informed by and situated within a wider body of the Māori wellbeing literature but we were able to draw upon the lived experiences of Māori people in their own words (making the process emergent and grounded) while interpreting and analysing with theory in mind and then engaging in member-checking to see whether these interpretations are valid and participating in collective meaning-making with others. These embodied and relationally embedded practices, common in Indigenous research contexts, were invaluable in ensuring our research was collective and thus aligned to Māori worldviews.

### 4.5. Practical Applications

We hope to see the Ngaruroro make it into the hearts and homes of everyday whānau, sparking critical thinking and reflection. We wish for this model to facilitate new dialogue, inspiring people to actively strengthen the connections, capacities, and lifestyle choices that they feel can improve their wellbeing.

Much like Te Whare Tapa Whā [9] and Te Wheke [21], the versatility of the Ngaruroro extends across various domains and can be applied across different levels. This scope includes educational, clinical, health promotion, policy-making, and research contexts. The Ngaruroro holds potential for offering insights at the whānau, community, and population levels, despite being initially designed to assist Māori individuals in identifying sources of wellbeing and serving as the foundation for a self-report measure.

We envisage the Ngaruroro being embraced by various peoples as a tool for connection and empowerment. Firstly, for Māori individuals on their cultural reconnection journeys, the model offers an accessible framework for how they may strengthen their wellbeing from a Māori perspective, especially in relation to wairua. Secondly, for peoples from other black, brown, Indigenous, and communities of colour living in Aotearoa, the Ngaruroro can serve as a bridge, recognising shared experiences and wellbeing needs. Sharing this model could foster belonging, understanding, and solidarity between non-Māori and Māori communities.

### 4.6. Important Caveats

#### 4.6.1. Wellbeing as Opposed to Waiora

The Ngaruroro is grounded in understandings of wellbeing for Māori people, and we distinguish ‘waiora’ (which is often translated as wellbeing) from the English concept of wellbeing, as waiora sits within the unique linguistic and conceptual landscape of te ao Māori. The model that has emerged out of this research does not therefore claim to speak to or represent waiora. As we continue to decolonise and indigenise our understandings and practices related to wellbeing, it is important that future research also investigates our ancestral knowledge related to waiora. There is scope for thorough examinations of our sources of mātauranga such as waiata (songs), haka (posture dances), mōteatea (chants), pūrākau (story), whakataukī (proverbs of unknown origin), whakatauākī (proverbs of known origin), and pepeha (tribal sayings). This search could provide valuable context, guidance, and insights for Māori researchers wishing to conduct further wellbeing research in accordance with Māori cultural traditions, worldviews, and belief systems. Doing so may involve collective theorising through wānanga, interviews with tohunga (Māori cultural experts), and analyses of historical documents.

#### 4.6.2. Wairua in Research

It is necessary to acknowledge that wairua is far too encompassing to be intellectualised and condensed into a mere theme or model. This notion is held by many wairua practitioners and is spoken to in a broader sense by Marsden [20] when he states “Māoritanga is a thing of the heart rather than the head” (p. 2). The boundless nature of wairua has resulted in most frameworks avoiding specific detail to allow for room for interpretation and subjective understandings, which protects the concept from being narrowed and misrepresented. But at the same time, having fewer descriptive details can leave people who do not possess a foundational understanding of wairua without guidance as to how they can start feeling their way into their Māoritanga. We believe that articulating points of entry to wairua is an important endeavour, with the provision that the appropriate caveats are made. The five wairua items from this model were derived from the interviews, a dedicated wānanga with the advisory rōpū, and detailed discussions with the research team during the data analyses. These items humbly represent a handful of ways in which wellbeing can be sourced in relation to wairua (see Marsden [20], Smith [48], and Valentine et al. [50] for a more comprehensive understanding of wairua). We believe that our inclusion of items for the wairua theme is an important contribution to the body of frameworks and models relating to Māori health and wellbeing.

#### 4.6.3. ‘Māori’ Level of Analysis

It is important to recognise that this research operates at the ‘Māori’ unit of analysis. Māori are not homogenous and are first peoples of various hapū and iwi collectives, each with unique mana, tikanga, kawa (protocols), histories, collective experiences of colonisation, and identities. We appreciate that there always have and always will be diverse Māori realities and by no means seek to homogenise Māori. It is also important to establish that models at this general ‘Māori’ level are still valuable, despite the current move towards more iwi- and hapū-centric frameworks and tools. This approach links back to Durie’s [43] argument that we need tools to measure wellbeing across all levels and units of analysis (individual, collective, population). Therefore, we have introduced Ngaruroro to add to the basket of Māori wellbeing tools, to be used when and where the context is appropriate.

#### 4.6.4. Diverse Māori Realities—Ngā Matatini Māori

Another important caveat is that the Ngaruroro is meant to be descriptive, not prescriptive. That is, the model was developed to help people better understand their wellbeing by identifying and describing different potential sources of wellbeing for them. The model does not prescribe any components of the model to be essential for someone to be well as a Māori person. We believe this viewpoint to be critical in the context of diverse Māori realities [9,12]. For example, connection to te reo Māori can be fundamental to the wellbeing of some Māori people but at the same time, not be for others. This distinction is crucial because models that prioritise like this can be used to marginalise Māori who have not had the opportunity to, for example, experience the nourishing feeling that speaking te reo Māori can bring them. We invite readers to become familiar with the concept of Māori cultural embeddedness to gain a more nuanced understanding of such issues [33,34,35].

### 4.7. Practical Limitations

This study is part of a wider PhD thesis project, which was conducted during the COVID-19 pandemic, which impacted several factors relating to budgets, methods, and timelines. These circumstances required agility, compassion, and flexibility from the research team and informants. As a result, several interviews had to be conducted online instead of face-to-face, and this situation made collective research approaches (i.e., focus groups) less viable. This situation was unfortunate as this research could have greatly benefitted from using more co-design and wānanga methods. These more collaborative community-driven approaches allow for much deeper insights and would have ultimately been more robust from a Kaupapa Māori perspective. The present study’s collective-focused approach has meant, however, that multiple sources have been able to inform the creation of this model, rather than just the interview data.

## 5. Conclusions

In this paper, we introduce a new model of Māori wellbeing, Ngaruroro, which adds to the growing number and variety of wellbeing models for Māori. The eight themes and forty-one items offer fresh, unique, and detailed insights as to what is important to the wellbeing of Māori people today and identify a variety of potential sources of wellbeing. The Ngaruroro describes wellbeing as the embodied and active process of being well in relation with one’s (1) here tāngata (social and familial ties), (2) te taiao (the environment), and (3) taonga tuku iho (cultural treasures) while doing what one can to make lifestyle choices that are conducive to the health of one’s (4) tinana (body) and (5) wairua (spirit) while cultivating a balanced (6) ngākau (inner-system), fulfilling (7) matea (core needs), and exercising one’s (8) mana (authority). Like the Ngaruroro River, our wellbeing is dynamic in how it naturally ebbs and flows, has times of activity and inactivity, emerges as the result of multiple interconnected streams of water, and exists within a holistic system of connections.

## Figures and Tables

**Table 1:** The members of the advisory rōpū and their iwi affiliations.

Name	Iwi Affiliations
Mike Ross	Ngāti Hauā
Veronica Thompson	Ngāti Kahungunu ki Wairarapa, Te Iwi Morehu
Stephanie Tibble	Rongomaiwahine, Rongowhakaata, Ngāti Kahungunu, Ngāti Hine
Natasha Tassell-Matamua	Te Āti Awa, Ngāti Makea kei Rarotonga
Kevin Shedlock	Ngāpuhi, Ngāti Porou, Whakatōhea

**Table 2:** The name and iwi affiliations of the participants from the first set of interviews.

Participant Name	Iwi Affiliations
Te Matahiapo Safari Hynes	Rangitāne, Ngāti Kahungunu
Victoria Bell	Kāi Tahu
Stevie-Jean Gear	Te Arawa, Ngāi Te Rangi
Monica Mercury	Ngāti Kahungunu, Te Iwi Morehu
Denis Grennell	Ngāti Maniapoto
Tau Huirama	Ngāti Tamanuipō, Ngāti Maniapoto
Mary Bennett	Ngaa Rauru Kiitahi, Ngāti Tūwharetoa, Te Āti Haunui-a-Pāpārangi
Clive Aspin	Ngāti Maru, Ngāti Whanaunga, Ngāti Tamaterā
Kahuwaero Katene	Ngāti Tūwharetoa, Ngāti Kahungunu

**Table 3:** The name and iwi affiliations of the participants from the second set of interviews.

Participant Name	Iwi Affiliation
Mikaere Paki	Ngāti Raukawa, Ngāti Apa, Ngāti Kauwhata
Connor Goggin	Rongomaiwahine, Ngāti Kahungunu
Chelsea Jacobs-Prescott	Ngāti Raukawa ki Te Tonga
Tohu Waetford Hekeata	Te Arawa
Gina Reiri	Ngāti Kahungunu ki Wairarapa
Tom Roa	Ngāti Maniapoto, Waikato
Paul Edwards	Te Whakatōhea, Ngāti Porou, Ngāi Tūhoe
Billy Corbett	Ngāpuhi, Ngāti Kahungunu, Te Rarawa
Rere-No-A-Rangi Pope	Ngāti Ruahine, Te Āti-Awa, Te Whakatōhea
Hana Kilford	Ngāti Kahungunu ki Wairoa
Pounamu Tipiwai-Chambers	Ngāti Kahungunu, Kāi Tahu, Ngāti Hineuru, Te Whānau-a-Apanui
Tere Gilbert	Te Āti Awa, Ngāti Kahungunu
Phillip Wilcox	Rongomaiwahine, Ngāti Rakaipaaka, Ngāti Kahungunu ki Te Wairoa
Ellie Rukuwai	Te Āti Haunui-a-Pāpārangi, Ngā Rauru, Ngāti Tūwharetoa
Annalisa Strauss-Hughes	Unknown

**Table 4:** The six items for the Here Tāngata theme and their English approximations.

Items	English Approximation
Whānau	Family
Hapū	Sub-tribe
Iwi	Tribe
Tīpuna	Ancestors
Hoa	Friends
Hapori	Communities

**Table 5:** The five items for the Tinana theme and their English approximations.

Items	English Approximation
Kai	Diet
Kori tinana	Physical activity
Moe	Sleep
Kai whakapiri	Substances that people use to feel a sense of connection or self-medicate
Kanohi kitea	Having your face physically seen

**Table 6:** The five items for the Here Tāngata theme and their English approximations.

Items	English Approximation
Kare-ā-roto	Emotions
Whakaaro	Thoughts
Waiaro	Attitudes
Aroha	Love
Pāmamae	Trauma, grief, deep pain

**Table 7:** The five items for the Wairua theme and their English approximations.

Items	English Approximation
Atua	Māori deities, ancestors of continued influence, god(s)
Wana	Exhilarating and breath-taking experiences
Wāhi wairua	Spaces that nurture your sense of wairua
Mahi aroha	Activities or work you do for passion, love, or service
Poipoi i te mauri	Nurturing the lifeforce of the beings, spaces, and things around you

**Table 8:** The five items for the Taiao theme and their English approximations.

Items	English Approximation
Ngahere	Bush and forests
Whenua	Land features
Wai tai	Bodies of salt water
Wai māori	Bodies of fresh water
Ngā rangi	Celestial bodies

**Table 9:** The five items for the Matea theme and their English approximations.

Items	English Approximation
Whai mātauranga	Acquiring knowledge
Tuku mātauranga	Passing on knowledge
Kainga	Housing
Pūtea	Money
Wā whakatā	Relaxation

**Table 10:** The five items for the Mana theme and their English approximations.

Items	English Approximation
Tū tangata	Stand in the fullness of who you are
Whiriwhiri	Power to decide how your life unfolds
Manaaki	Uplifting, caring for, and being hospitable to others
Whakatere	Navigate challenges in life
Tū toa	Stand confident, accomplished, or capable in a skill or area

**Table 11:** The five items for the Taonga Tuku Iho theme and their English approximations.

Items	English Approximation
Te reo Māori	Māori language
Tikanga Māori	Māori customary protocols and practices
Mātauranga Māori	Traditional and contemporary Māori knowledge
Uaratanga Māori	Māori values
Tūrangawaewae	Traditional and contemporary places of belonging

**Table 12:** The eight themes, their relevant descriptions, and items.

Theme	Description	Items
Here Tāngata	Connection to social and familial ties	Whānau
		Hapū
		Iwi
		Tīpuna
		Hoa
Tinana	Lifestyle choices related to the tinana and physical health	Kai
		Kori tinana
		Moe
		Kanohi kitea
		Kai whakapiri
Ngākau	Capacities related to the ‘inner-world’	Kare-ā-roto
		Whakaaro
		Waiaro
		Aroha
		Pāmamae
Wairua	Lifestyle choices related to spirit and interconnectedness	Atua
		Wana
		Wāhi wairua
		Mahi aroha
		Poipoi i te mauri
Taiao	Connection to the environment	Ngahere
		Whenua
		Wai tai
		Wai māori
		Ngā rangi
Matea	Capacities to meet core needs	Whai mātauranga
		Tuku mātauranga
		Kainga
		Pūtea
		Wā whakatā
Mana	Capacities related to exercising mana	Tū tangata
		Whiriwhiri
		Manaaki
		Whakatere
		Tū toa
Taonga Tuku Iho	Connection to cultural treasures	Te Reo Māori
		Tikanga Māori
		Mātauranga Māori
		Uaratanga Māori
		Tūrangawaewae

## Data Availability

The qualitative data presented in this study will not be shared to maintain the participants’ governance over their data.
